# Comparative effectiveness of different therapies for treating striae distensae: A systematic review and network meta-analysis

**DOI:** 10.1097/MD.0000000000022256

**Published:** 2020-09-25

**Authors:** Haishan Lu, Jian Guo, Xudong Hong, Aifen Chen, Xudong Zhang, Shengxian Shen

**Affiliations:** Department of Dermatology, PLA 903 Hospital, Hangzhou, Zhejiang, China.

**Keywords:** clinic treatment, meta-analysis, randomized controlled trail, striae distensae

## Abstract

**Background:**

Striae distensae (SD) are common and aesthetically undesirable dermal lesions. The aim of this study is to comprehensively evaluate the effectiveness of different therapies in treating striae distensae using network meta-analysis.

**Methods:**

A systematic search of electronic databases up to December 1, 2019 was conducted. Randomized controlled trails (RCTs) examining the effectiveness of different methods in treating striae distensae were included. The primary outcomes are clinical effective rate and patient's satisfaction degree. Risk of bias was assessed by the Cochrane risk of bias tool. Network meta-analysis was based on Bayesian framework.

**Results:**

Fourteen trails that met the criteria with 651 subjects were included. The results of the network meta-analysis show that topical tretinoin combined bipolar radiofrequency showed the highest probability of being the best method to improve the clinical effectiveness and patient satisfaction rate of treating SD (84.5% and 95.7% respectively), closely followed by bipolar radiofrequency (75.3% and 84.3% respectively). Among laser treatment, CO_2_ fractional laser is superior to other lasers in the clinical effectiveness and patient satisfaction (72.0% and 58.1% respectively). Statistics showed the topical tretinoin was the worst-performing option in improving the clinical effectiveness and patient satisfaction rate of SD treatment (5.4% and 5.1% respectively).

**Conclusion:**

Based on the results of network meta-analysis, we recommend treating striae distensae with bipolar radio frequency combined topical tretinoin. The commonly used CO_2_ fractional laser can be considered as alternative treatment candidate. Additional large-scale RCTs are necessary to obtain more precise estimates of their relative efficacy.

## 1 Introduction

Striae distensae (SD) or stretch marks are common and aesthetically undesirable dermal lesions, which closely related to rapid skin stretching, hormonal changes, and genetic factors.^[1]^ Manifested as atrophic linear plaques, SD initially present as flattened or slightly raised pink or red scars (striae rubra) before subsequently turning into paler, flat, and permanent (striae alba).^[2]^ SD generally appears along the cleavage lines in the abdomen, hips, thighs, and breasts, commonly developing during pubertal growth spurts and pregnancy, and associated with Cushing syndrome and oral or topical corticosteroid use.^[3]^ The pathophysiological mechanism of SD involved in an inflammatory reaction and elastolysis arising from the release of elastase from mast cells.^[2,4]^ Similar to scars, SD histologically is characterized by thinning of the overlying epidermis, loss of rete ridges, densely packed collagen bundles aligned parallel to the reticular dermis and atrophic skin.^[1,5]^ Although not regarded as a disease, SD results in substantial psychological discomfort and cosmetic concern for women.^[6]^

Several treatments methods, such as topical agents, microdermabrasion, laser therapies, light therapies, needling therapy, radio frequency (RF) devices, platelet-rich plasma (PRP) injection, have been advocated with variable efficacy.^[7]^ However, there are no clear ranking of these therapies in terms of their clinical effectiveness and patient satisfaction. With technological advances in aesthetic medicine, an updated quantitative comparative research was needed to aid in clinical decision making.

To tackle this problem, network meta-analysis that is a statistical tool of quantifying evidence from a network of multiple randomized controlled trails (RCTs) can be employed.^[8]^ This method can simultaneously compare multiple competing interventions that have no head-to-head trails available in a single statistical model.^[9]^ The aims of our study are to conduct a systematic review and network meta-analysis of RCTs to comprehensively assess the clinical effectiveness and patient satisfaction of different methods for treating striae distensae.

## 2 Methods

According to Preferred Reporting Items for Systematic Reviews and Meta-Analysis (PRISMA) statement and PRISMA Extension Statement for Network Meta-Analysis, a systematic review and network meta-analysis was performed.^[10]^ The data and information used in our study was from previously published clinical trials; therefore, ethical approval from Ethics Committee and Institutional Review Board was not required.

### 2.1 Search strategy and selection criteria

We searched PubMed, Cochrane Library, and EMBASE online databases by 2 independent authors (JG and DX). The literature search was performed until December 1, 2019, and all published RCTs were included in the review. The following key terms were selected under Medical Subject Headings for the search: “striae distensae” or “stretch marks” or “striae rubra” or “striae alba” or “striae gravidarum” and “randomized controlled trails.” To avoid the potential omission of studies, we searched additional databases, such as opengrey.eu for gray literature. We also manually screened reference lists of previous systematic reviews. Two reviewers (AC and XZ) independently reviewed the titles and abstracts of all the studies, and the studies that satisfied the inclusion criteria were retrieved for assessments. Disagreements between the reviewers were resolved by consensus. Duplicates were removed using Endnote X9 (Thomson Reuters Co, New York).

### 2.2 Eligibility criteria

#### 2.2.1 Types of studies

Randomized controlled clinical trials for treating striae distensae were included. Studies should evaluate at least 2 therapies. Trials were excluded if they did not provide validated therapeutic protocols and did not report the results of the clinical effectiveness and patient satisfaction.

#### 2.2.2 Types of interventions

Interventions included topical agents, microdermabrasion, laser therapies, light therapies, needling therapy, radio frequency RF devices, PRP injection, and combined therapies. The therapeutic methods were compared with each other.

#### 2.2.3 Types of outcomes

We selected the clinical effectiveness and patient satisfaction rate as the outcomes of this study. Interventions included topical agents, microdermabrasion, laser therapies, light therapies, needling therapy, radio frequency RF devices, PRP injection, and combined therapies. The therapeutic methods were compared with each other. The clinical effectiveness was analyzed using photographic materials by 2 dermatologists blinded to the study group. Evaluators used a quartile grading scale of 0 = no improvement, 1 = 1% to 25% (mild) improvement, 2 = 26% to 50% (moderate) improvement, 3 = 51% to 75% (good) improvement, and 4 = 76% to 100% (excellent) improvement. Each participant was asked to rate overall satisfaction as 0 = unsatisfied, 1 = slightly satisfied, 2 = moderately satisfied, 3 = satisfied, or 4 = very satisfied.

### 2.3 Data extraction and quality assessment

Two reviewers (AC and XZ) independently extracted the data using a predesigned strategy. The extracted information included the participant characteristics, interventions, intervention time, outcome measures, funding, and conflicts of interests. Then, the data were integrated. Disagreements within the included study were resolved through discussion. If an agreement could not be reached, a third reviewer was consulted. Two reviewers (JG and XDH) independently assessed the risk of bias using the Cochrane Collaboration Risk of Bias Tool (Review Manager, V.5.2), which involving random sequence generation, allocation concealment, blinding, incomplete outcome data, selective reporting, and other biases. We also assessed the risk of bias across trials. If more than 50% of the information was from trials at a low risk of bias, the domain was judged to be at a low risk of bias. Similarly, if most information was from RCTs with an unclear/high risk of bias, the domain was considered to be at an unclear/high risk of bias.

### 2.4 Statistical analysis

Network meta-analysis using Bayesian-network method was applied to synthesize evidence for the primary outcome. This approach estimated relative effects of multiple treatments by fitting generalized linear model under Bayesian framework. Heterogeneity (*I*^2^) was evaluated to determine variability between the included studies. *I*^2^ value of 25% was defined as low heterogeneity, 50% as moderate heterogeneity, and 75% as high heterogeneity. Consistency of results from direct and indirect evidence was analyzed using the node-splitting analysis of inconsistency. All statistical analyses were conducted using STATA version 16.0.

### 2.5 Inconsistency analysis and sensitivity analysis

Model inconsistency was assessed using the node-splitting method. If the *P* value was smaller than .05, then an inconsistency was considered as detected. The node-splitting models were generated via the STATA 14.0. A sensitivity analysis was conducted to test the influence of low-quality studies. If the result of rank probability changes slightly, indicating that the results were credible.

## 3 Result

### 3.1 Search results

**Figure 1:**
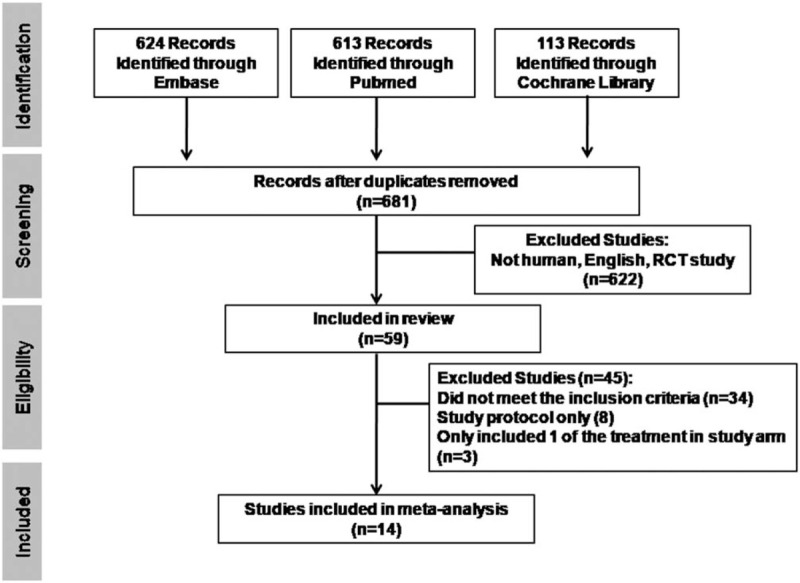
Flowchart for literature search result. RCT = randomized controlled trial.

Our search strategy yielded a total of 1350 potentially relevant articles. Of these, 59 trials were included based on the titles and abstracts. After careful full-text screening, 45 articles were discarded for the reasons listed in Figure 1. Fourteen RCTs^[11–24]^ met the inclusion criteria, involving nonablative laser, CO_2_ fractional laser, Nd:YAG laser, intense pulsed light, topical tretinoin, carboxytherapy, platelet-rich plasma injection, microdermabrasion, bipolar radiofrequency, microneedling, and combined treatment methods. The characteristics of the included trials are presented in Table 1. The total numbers of participants in these studies were 651.

**Table 1:**
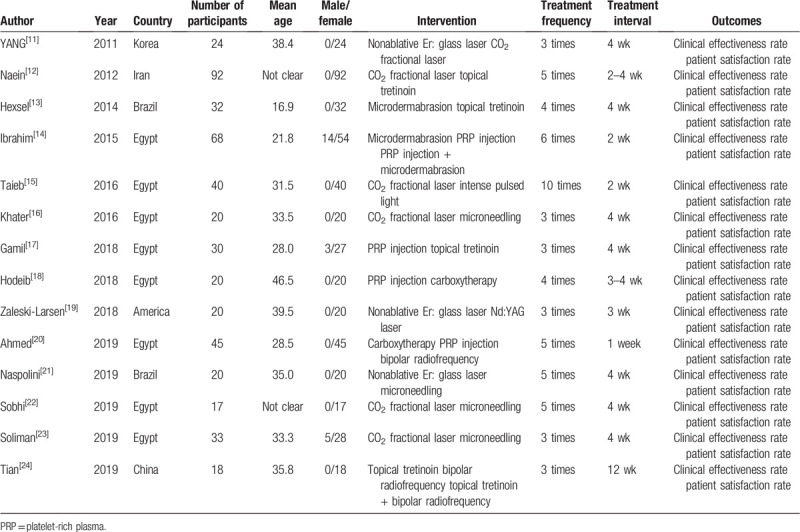
Characteristics of the included studies.

### 3.2 Risk of bias and quality assessment

**Figure 2:**
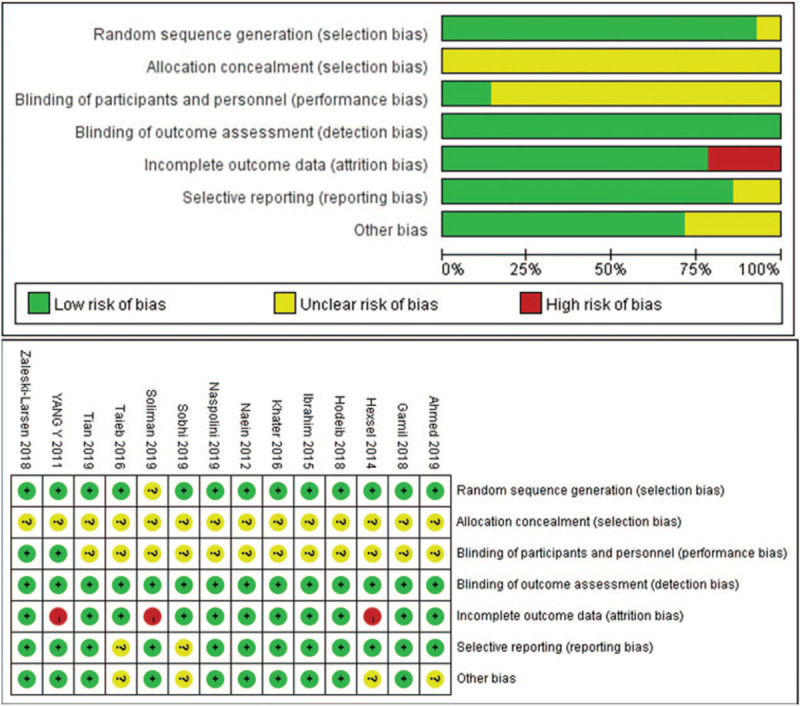
Risk of bias graph (upper) and summary (lower).

Quantification of the risk of bias assessment is presented in Figure 2. A random sequence was generated in 13 trials, suggesting the risk of bias in randomization was low. All the studies had unclear information about the methods used to conceal the allocation, therefore, we considered that the risk of bias was unclear for the domain of allocation concealment. The outcomes evaluators were successfully blinded in all included trials, and the risk of bias in this domain was judged to be low. However, the participants and personnel were unclearly blinded in most trials (n = 12, 85.7%). As for the incomplete outcome data element, there was a low risk of bias because most studies reported complete data (n = 11, 78.6%). There was also a low risk of selective outcome reporting because 12 of the studies had a low risk of bias in this domain (85.7%). In addition, the risk of other biases was also low (n = 10, 71.4%). Overall, the certainty evidence of study was moderate.

### 3.3 Results of the network meta-analysis

**Figure 3:**
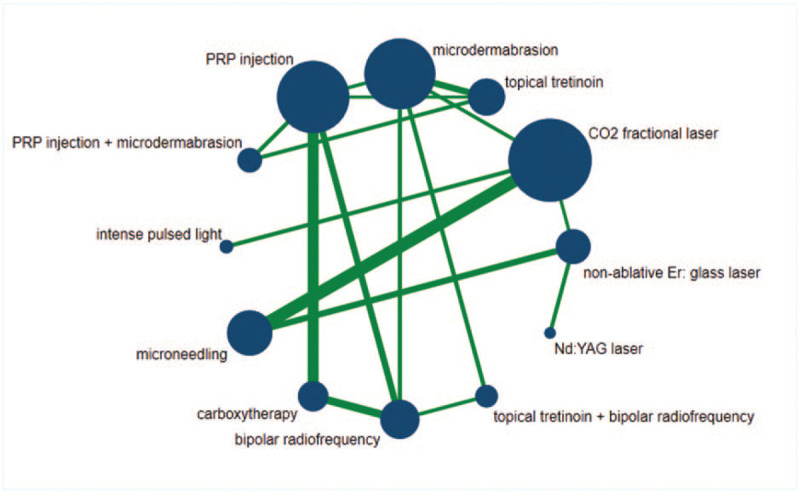
Network of comparisons of nonablative laser, CO_2_ fractional laser, Nd:YAG laser, intense pulsed light, topical tretinoin, carboxytherapy, platelet-rich plasma (PRP) injection, microdermabrasion, bipolar radiofrequency, microneedling, and combined treatment methods for treating SD. Note: The size of the circle represents the number of participants, and the thickness of the edge represents the number of studies.

The network graph was built via STATA shown in Figure 3. The size of the circle represents the number of participants, and the thickness of the edge represents the number of studies. All potential comparisons were calculated and presented as ORs and 95% CrIs. The results are listed in Table 2, and the significant differences are shaded.

**Table 2:**
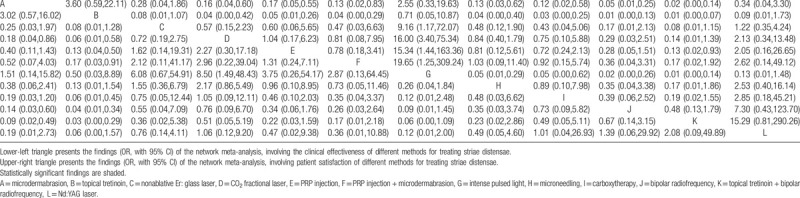
Results (odd radio, with 95% confidence interval) of the network meta-analysis.

In the clinical effectiveness aspect, CO_2_ fractional laser, bipolar radiofrequency, topical tretinoin + bipolar radiofrequency significantly yielded better outcomes than microdermabrasion; CO_2_ fractional laser, PRP injection, PRP injection + microdermabrasion, carboxytherapy, bipolar radiofrequency, topical tretinoin + bipolar radiofrequency were significantly more superior than topical tretinoin; CO_2_ fractional laser was significantly better than intense pulsed light. No significant differences were found in other comparisons (Table 2, lower-left triangle).

With regard to patient satisfaction rate, CO_2_ fractional laser, PRP injection, PRP injection + microdermabrasion, microneedling, carboxytherapy, bipolar radiofrequency, topical tretinoin + bipolar radiofrequency significantly yielded better outcomes than microdermabrasion and topical tretinoin; nonablative Er: glass laser, CO_2_ fractional laser, PRP injection, PRP injection + microdermabrasion, microneedling, carboxytherapy, bipolar radiofrequency, topical tretinoin + bipolar radiofrequency were significantly more superior than intense pulsed light; topical tretinoin + bipolar radiofrequency was significantly better than PRP injection. No significant differences were found in other comparisons (Table 2, upper-left triangle).

### 3.4 Rank probability based on SUCRA

**Figure 4:**
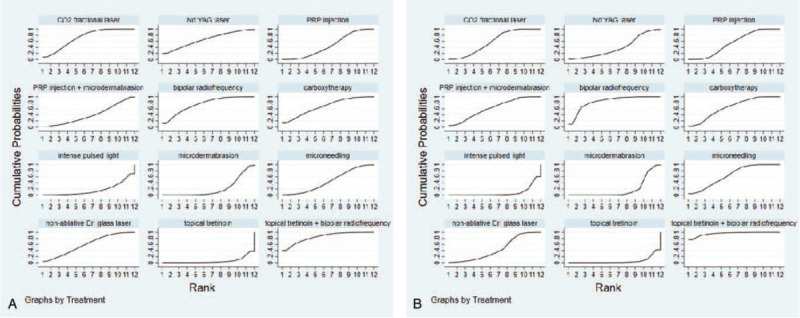
SUCRA of nonablative laser, CO_2_ fractional laser, Nd:YAG laser, intense pulsed light, topical tretinoin, carboxytherapy, platelet-rich plasma (PRP) injection, microdermabrasion, bipolar radiofrequency, microneedling, and combined treatment methods for improving the clinical effectiveness (A) and patient satisfaction rate (B) of treating SD. Note: The area under the curve represents the cumulative rank probability of each treatment. The larger the area, the better the cumulative rank probability. SUCRA = surface under the cumulative ranking curve.

The ranking probability in terms of the clinical effectiveness and patient satisfaction rate is illustrated in Figure 4. Larger areas under the surface under cumulative ranking curve represent better effectiveness. Topical tretinoin combined bipolar radiofrequency showed the highest probability of being the best method to improve the clinical effectiveness and patient satisfaction rate of treating SD (84.5% and 95.7% respectively), closely followed by bipolar radiofrequency (75.3% and 84.3% respectively). Among laser treatment, CO_2_ fractional laser is superior to other lasers in the clinical effectiveness and patient satisfaction (72.0% and 58.1% respectively). Statistics showed the topical tretinoin was the worst-performing option in improving the clinical effectiveness and patient satisfaction rate of SD treatment (5.4% and 5.1% respectively).

### 3.5 Inconsistency analysis

Inconsistency in network meta-analysis denotes the consistency between direct and indirect evidence for each intervention. The node splitting analysis showed all clinical trials were consistent between direct and indirect comparisons (*P* > .05).

### 3.6 Sensitivity analysis

After excluding 1 low-quality study (Soliman et al), the SUCRA changed slightly, although no change occurred in the rank probabilities, indicating that the results of the network meta-analysis are robust.

## 4 Discussion

### 4.1 Main findings

In this systematic review and network meta-analysis, we combined direct and indirect evidence from 14 studies including 651 participants. Our network meta-analysis compared the clinical effectiveness and patient satisfaction of microdermabrasion, topical tretinoin, nonablative Er: glass laser, CO_2_ fractional laser, PRP injection, PRP injection combined microdermabrasion, IPL, microneedling, carboxytherapy, bipolar radiofrequency, topical tretinoin combined bipolar radiofrequency, Nd:YAG laser therapy for the treatment of SD. With moderate certainty, our study indicated that topical tretinoin combined bipolar radiofrequency and bipolar radiofrequency had the highest probability of providing the best outcome in terms of clinical effectiveness and patient satisfaction. Topical tretinoin was the worst-performing option in improving the clinical effectiveness and patient satisfaction rate of SD treatment.

### 4.2 Agreements and disagreements with other studies

A previous review reported that 1540-nm nonablative fractionated laser was a worthy first-line modality for the treatment of SD,^[3]^ based on clinical and anecdotal experience, which was inconsistent with our meta-analysis. On the one hand, our study indicated that bipolar radiofrequency was superior to laser treatment. On the other hand, CO_2_ ablative fractional laser provided better outcome than nonablative Er: glass laser in the clinical effectiveness and patient satisfaction of SD treatment.

Bipolar radiofrequency can pass through the skin, and generate heat due to the resistance of the skin, acting on the deep dermis and fibrosis septum, and sometimes fascia, causing the contraction of existing collagen and synthesis of new collagen.^[25]^ It accelerates the healing by means of fractional mode, so as to improve the safety of the treatment, similar to the mechanism of fractional lasers.^[26]^ The bipolar radiofrequency system used in the included study can deliver energy in a fractional manner, and generates an array of micro-thermal zones of thermal injury in the epidermis and dermis. We assumed that the bipolar fractional radiofrequency system disrupts the stratum corneum, causing microchannels in the epidermis to alter the skin permeability so as to promote topical tretinoin penetration. The combined therapy is indicated to have a synergistic effect, which accelerates collagen synthesis and fibroblast activity.^[24]^ Consistent with our study, the majority of trials evaluating RF for the treatment of SD have reported significant improvements, with few side effects including erythema and edema. In addition, some studies have also demonstrated subjective improvement of SD via bipolar RF combined with and ablative CO2 laser and bipolar RF combined with PRP injection.^[25–27]^ The participants with Fitz-partrick Skin Types IV and V reported on adverse event, indicating that bipolar RF may be considered as a potential and safe therapeutic option for SD patients with skin of color.^[2]^

A variety of laser parameters have been studied either alone or in combination with other modalities for the treatment of SD, among which the effectiveness of nonablative fractional lasers versus ablative fractional lasers was controversial.^[5,28]^ Our study found that CO_2_ ablative fractional laser was significantly superior to nonablative Er: glass laser. However, it was based on only 1 RCT directly comparing CO_2_ ablative fractional laser and nonablative Er: glass laser.

Numerous studies reported that tretinoins was able to increase tissue collagen I levels through stimulation of fibroblasts so as to improve the appearance of early SD.^[29,30]^ In our study, compared with other methods, the topical tretinoin was the worst-performing option in improving the clinical effectiveness and patient satisfaction rate of SD treatment. However, when topical tretinoin combined with bipolar RF, this strategy showed the highest probability of being the best method to improve the clinical effectiveness and patient satisfaction rate of treating SD. Our study suggested that topical tretinoin can be used to treat SD combined with other treatment methods.

### 4.3 Strengths and limitations

There were several strengths in our net-work meta-analysis. Firstly, our study was the first network meta-analysis of RCTs to comprehensively assess the clinical effectiveness and patient satisfaction of different methods for treating striae distensae. Secondly, we conducted a comprehensively searched for literature, with 2 independent reviewers assessing quality, to reduce any potential bias. Thirdly, our network model consisted of a closed loop, which allowed for the assessment of inconsistency for both direct and indirect evidence. More importantly, all the trials included were RCTs, which were the highest level of evidence, thus our results should strongly reflect the true clinical effectiveness of these methods. In addition, the node-splitting analysis showed all clinical trials were consistent between direct and indirect comparisons, indicating our analysis was based on consistent evidence.

**Figure 5:**
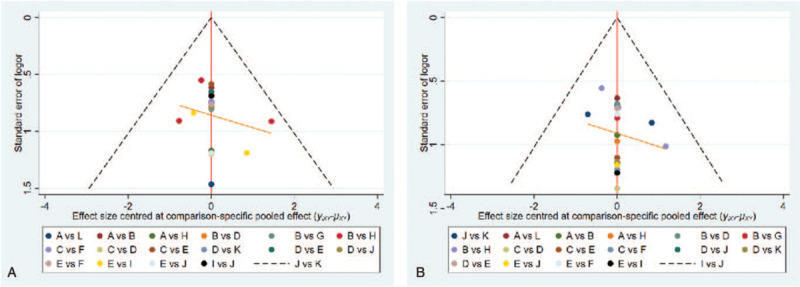
Funnel plots of the included studies involving the clinical effectiveness (A) and patient satisfaction rate (B) of treating SD.

Nevertheless, this study also has limitations. First, some treatments were presented in only 1 study. Thus, more high-quality RCTs were needed in the future to corroborate these results. Second, the participants were unclearly blinded in most trials, which may result in participant bias. Third, the English restriction during the search can possibly affect the comprehensiveness of the search strategy. Finally, there may be a publication bias because all the articles reported positive results of their clinical trials. But our funnel plots showed the publication bias was low (Fig. 5).

## 5 Conclusion

Our network meta-analysis compared the clinical effectiveness and patient satisfaction of 14 modalities for treating striae distensae. With moderate certainty, our study indicated that topical tretinoin combined bipolar radiofrequency and bipolar radiofrequency had higher probability of providing the better outcome in terms of clinical effectiveness and patient satisfaction. However, topical tretinoin only was the worst-performing option in improving the clinical effectiveness and patient satisfaction rate of SD treatment. Additional large-scale RCTs are necessary to obtain more precise estimates of their relative efficacy.

## Acknowledgments

The authors gratefully acknowledge the financial support from “Zhejiang Provincial Natural Science Foundation Basic Public Welfare Research Project”. (LGF18H110003).

## Author contributions

**Conceptualization:** Haishan Lu, Jian Guo, Shengxian Shen.

**Formal analysis:** Haishan Lu, Jian Guo, Xudong Hong, Aifen Chen.

**Funding acquisition:** Haishan Lu, Aifen Chen, Xudong Zhang.

**Investigation:** Haishan Lu, Jian Guo, Aifen Chen.

**Resources:** Jian Guo, Shengxian Shen.

**Supervision:** Shengxian Shen, Xudong Zhang.

**Writing – original draft:** Haishan Lu, Jian Guo.

**Writing – review & editing:** Haishan Lu, Xudong Hong, Shengxian Shen.
